# Impact of Alcohol on Bone Health in People Living With HIV: Integrating Clinical Data From Serum Bone Markers With Morphometric Analysis in a Non‐Human Primate Model

**DOI:** 10.1002/jbm4.10703

**Published:** 2022-11-28

**Authors:** Alexandra Denys, Allison Norman, Daniel S Perrien, Larry J Suva, Liz Simon, Lee S McDaniel, Tekeda Ferguson, Kim Pedersen, David Welsh, Patricia E Molina, Martin JJ Ronis

**Affiliations:** ^1^ Department of Pharmacology and Experimental Therapeutics Louisiana State University Health Sciences Center New Orleans LA USA; ^2^ Division of Clinical Pharmacology in the Department of Medicine Vanderbilt University Medical Center Nashville TN USA; ^3^ Department of Veterinary Physiology and Pharmacology, College of Veterinary Medicine and Biomedical Sciences Texas A&M University College Station TX USA; ^4^ Comprehensive Alcohol Research Center Louisiana State University Health Sciences Center New Orleans LA USA; ^5^ Department of Physiology Louisiana State University Health Sciences Center New Orleans LA USA

**Keywords:** NUTRITION, BIOCHEMICAL MARKERS OF BONE TURNOVER, BONE MODELING AND REMODELING, OSTEOPOROSIS, DISEASES AND DISORDERS OF/RELATED TO BONE

## Abstract

People living with HIV (PLWH) represent a vulnerable population to adverse musculoskeletal outcomes due to HIV infection, antiretroviral therapy (ART), and at‐risk alcohol use. Developing measures to prevent skeletal degeneration in this group requires a grasp of the relationship between alcohol use and low bone mass in both the PLWH population and its constituents as defined by sex, age, and race. We examined the association of alcohol use with serum biochemical markers of bone health in a diverse cohort of PLWH enrolled in the New Orleans Alcohol Use in HIV (NOAH) study. To explore the effects of alcohol on bone in the context of HIV and ART and the role of estrogen, we conducted a parallel, translational study using simian immunodeficiency virus (SIV)^+^/ART^+^ female rhesus macaques divided into four groups: vehicle (Veh)/Sham; chronic binge alcohol (CBA)/Sham; Veh/ovariectomy (OVX); and CBA/OVX. Clinical data showed that both osteocalcin (Ocn) and procollagen type I N‐propeptide (PINP) levels were inversely associated with multiple measures of alcohol consumption. Age (>50 years) significantly increased susceptibility to alcohol‐associated suppression of bone formation in both female and male PLWH, with postmenopausal status appearing as an additional risk factor in females. Serum sclerostin (Scl) levels correlated positively with measures of alcohol use and negatively with Ocn. Micro‐CT analysis of the macaque tibias revealed that although both CBA and OVX independently decreased trabecular number and bone mineral density, only OVX decreased trabecular bone volume fraction and impacted cortical geometry. The clinical data implicate circulating Scl in the pathogenesis of alcohol‐induced osteopenia and suggest that bone morphology can be significantly altered in the absence of net change in osteoblast function as measured by serum markers. Inclusion of sophisticated tools to evaluate skeletal strength in clinical populations will be essential to understand the impact of alcohol‐induced changes in bone microarchitecture. © 2022 The Authors. *JBMR Plus* published by Wiley Periodicals LLC on behalf of American Society for Bone and Mineral Research.

## Introduction

The advent of antiretroviral therapy (ART) has allowed people living with HIV (PLWH) to live longer, in turn unmasking an increased risk for age‐associated metabolic disturbances in this population.^(^ ^1^ ^)^ The musculoskeletal system is one of the affected organ systems, with several studies demonstrating that PLWH have a 2‐ to 3.5‐fold increased risk of fracture compared with the general population.^(^ ^2^, ^3^, ^4^ ^)^ This risk is multifactorial, and the underlying mechanisms are poorly understood. Alcohol use disorder (AUD), which afflicts 30% of PLWH worldwide,^(^ ^5^ ^)^ is recognized as a leading risk factor for the development of osteoporosis,^(^ ^6^, ^7^ ^)^ a disease characterized by low bone mineral density (BMD) and bone fragility.^(^ ^8^ ^)^ Further, some classes of ART medications have been shown to acutely decrease BMD,^(^ ^9^, ^10^, ^11^, ^12^ ^)^ and HIV infection itself can disrupt bone homeostasis by promoting osteoclastogenesis.^(^ ^13^, ^14^, ^15^ ^)^ Whether the altered physiological state of HIV/ART alters or aggravates alcohol effects on bone and whether more so in particular groups is unknown. Considering the profound disability associated with fractures,^(^ ^16^, ^17^ ^)^ predictive markers of poor bone health and a better understanding of how high‐risk alcohol use in the context of HIV‐related comorbidities contributes to diminished bone mass and quality are urgently required.

To address this need and study the relationship between alcohol use and the exacerbation of HIV‐related comorbidities, the Comprehensive Alcohol‐HIV/AIDS Research Center (CARC) at the LSUHSC in New Orleans is conducting an ongoing, prospective translational study enrolling in‐care PLWH over the age of 18 years.^(^ ^18^ ^)^ Patients in this cohort have a wide range of alcohol use, which is thoroughly characterized using a biochemical marker and validated self‐reported measures.^(^ ^19^, ^20^ ^)^ We previously analyzed serum from a representative 40‐patient sample from this study and published early findings that linked at‐risk alcohol use with decreased serum osteocalcin (Ocn).^(^ ^21^ ^)^ This preliminary study did not allow us to contrast the responses between men and women of the cohort, which current evidence suggests are considerably different in the absence of HIV infection.^(^ ^22^, ^23^ ^)^ The field also attributes special consideration for postmenopausal women, the most vulnerable group to osteoporosis, as menopause is tied to sharp declines in the levels of bone‐protective estrogen.^(^ ^22^, ^24^ ^)^

With the goal to examine the contribution of these biological variables on the bone response to alcohol, we analyzed all (*n* = 356) samples obtained at baseline from the cohort for Ocn and procollagen type I N‐terminal propeptide (PINP) available at the beginning of this study. This size allowed us to determine the dose–response curve of alcohol consumption and inhibition of bone formation for the entire study and for different groups obtained by stratification of biological variable (sex, age, race). In women, the cut‐off age of 50 years also serves as a surrogate for menopausal status: 51 years is the average age of menopause onset in the United States and HIV accelerates onset.^(^ ^25^, ^26^ ^)^ To obtain mechanistic insights into the relationship between alcohol consumption and bone formation, we also measured blood levels of other proteins proposed to be indicative of musculoskeletal health, namely sclerostin (Scl)^(^ ^27^, ^28^, ^29^ ^)^ and dickkopf‐1 (DKK1).^(^ ^30^, ^31^ ^)^

We additionally performed a long‐term chronic binge alcohol (CBA) translational study in non‐human primate (NHP) rhesus macaques to directly evaluate bone. Half of the macaques were treated with CBA (versus vehicle), and all were subjected to simian immunodeficiency virus (SIV) infection and ART to systematically examine the cumulative effects of these treatments. Because we expected the most profound difference in response to alcohol to exist between younger (premenopausal) and older (postmenopausal) women in our clinical cohort due to estrogen status, we used female animals and generated two additional groups per diet, ovariectomized (OVX) and Sham, each undergoing the SIV‐ART treatment. In adult animals, ovariectomy is accepted to replicate the postmenopausal status.^(^ ^22^, ^32^ ^)^ We hypothesized that ovariectomy (OVX) in the context of SIV and ART would exacerbate the effects of CBA on suppression of bone formation, which would be revealed at the level of both serum markers and bone via micro‐CT analysis.

## Subjects and Methods

### The New Orleans Alcohol Use in HIV (NOAH) cohort

The NOAH study is an ongoing, longitudinal investigation of AUD, HIV/AIDS, and ART in aging and exacerbated comorbid conditions in an underserved cohort of PLWH.^(^ ^18^ ^)^ Initially, 365 HIV‐infected individuals under care at an HIV specialty clinic were enrolled in the cohort. It is composed predominantly of middle‐aged African American men who are virally suppressed and not critically CD4^+^ T‐cell immunodeficient, likely attributable to a high combination ART adherence rate. Approximately 84% to 87% of participants report ART adherence above 90%.^(^ ^33^ ^)^ The study population was oversampled toward individuals with Alcohol Use Disorder Identification Test (AUDIT) scores ≥8, suggestive of potentially hazardous drinking habits. Exclusion criteria included acute illness within the preceding 6 weeks (defined by unscheduled health care utilization for a new or exacerbated illness), nonprophylaxis administration of antibiotics, pregnancy, or acute intoxication. The Louisiana State University Health Sciences Center, New Orleans Human Research Protection Program and Institutional Review Board approved and oversaw the study. Further study and data collection details are described in Welsh and colleagues.^(^ ^18^ ^)^ Of 365 serum samples, 356 were available for analysis.

### Alcohol use assessment in the clinical cohort

Alcohol use was assessed using the AUDIT. The AUDIT is a 10‐item tool developed by the World Health Organization with an overall score ranging from 0 to 40,^(^ ^34^ ^)^ whereas AUDIT‐C represents its first three items and is used as a screen for hazardous drinking behavior. A timeline followback (TLFB) calendar was used to assess quantity and frequency of alcohol use and drinking patterns over the previous 14 and 30 days. For TLFB, participants were interviewed with prompts to report days in which alcoholic beverages were consumed. Total grams of alcohol consumed were tabulated by calculating the total standard alcohol drinks consumed by day and type (beer, wine, or liquor), which was then used to calculate total grams (g) of alcohol consumed each day; 14 g of alcohol was equated to one standard drink. Whole blood PEth, a highly reliable blood test for alcohol use, was used as a biomarker to assess alcohol use over the previous 3 to 4 weeks.^(^ ^35^ ^)^ Dried blood spot samples were analyzed by the United States Drug Testing Laboratories, Inc. (Des Plains, IL, USA). A measure of ≥8 ng/mL was considered a positive test and a measure of >250 ng/mL was categorized as alcohol misuse. PEth information was missing for 6 patients.

### Animal characteristics and study design

Adult (6‐ to 9‐year‐old) female Indian rhesus macaques (*Macaca mulatta*) were randomized based on in vitro SIV infectivity kinetics to either chronic binge alcohol (CBA) or isovolumetric water (VEH) groups as previously described.^(^ ^36^ ^)^ Macaques were administered alcohol at a concentration of 30% (w/v) in water (30 minutes infusion; 5 days/week; 12–15 g/kg/week) intragastrically. Peak plasma alcohol concentrations averaged 50 to 60 mM (~0.23% blood alcohol content) 2 hours after alcohol initiation. After 3 months of CBA/VEH administration (pre‐SIV), animals were inoculated intravaginally with SIVmac251. CBA/VEH administration continued throughout the study. Animals identified as “slow progressors” were selected to provide for a more homogeneous cohort of animals. This approach has been used to decrease variability between the experimental groups and increase the ability to evaluate the impact of alcohol as a cofactor of disease progression in our sample size. Selection was based on low capacity of in vitro infection by SIV_ΔB670_ of peripheral blood mononuclear lymphocytes (PBML).^(^ ^37^ ^)^ At viral set point (2.5 months post‐SIV infection), all macaques were initiated on daily subcutaneous injections of 20 mg/kg of tenofovir (9‐[2‐phosphonomethoxypropyl] adenine, PMPA) and 30 mg/kg of emtricitabine (FTC), provided by Gilead Sciences Inc. (Foster City, CA, USA). This drug combination and dose suppresses viral load and results in minimal toxicity in normal healthy macaques from infancy to adulthood and does not result in liver or renal toxicity in SIV‐infected macaques.^(^ ^38^ ^)^ One month post‐initiation of ART, animals were randomized to either OVX or SHAM surgeries for a total of four treatment groups: VEH/Sham (*n* = 8), VEH/OVX (*n* = 7), CBA/Sham (*n* = 7), and CBA/OVX (*n* = 7). Food consumption was monitored, and the macaques were provided with Boost/Ensure supplement or PRIMA‐Burger (Labserv) if there was a 10% reduction in food intake. Physical exams performed every week included monitoring body weight and measures of general health. Eight months after OVX or SHAM (study endpoint), after an overnight fast, all macaques were euthanized according to the American Veterinary Medical Association's guidelines.

### Serum analysis

Serum from NOAH PLWH was collected at baseline by centrifugation and stored at −80°C. All 356 human serum samples were analyzed using commercially available kits for total Ocn (catalog no. KAQ1381; Invitrogen, Waltham, MA, USA) and PINP (catalog no. ab210966; Abcam, Cambridge, MA, USA) according to the manufacturer's instructions. Samples were then randomly sampled and analyzed as follows: 157 were analyzed for Scl (TE1023‐HS; TECOmedical, Sissach, Switzerland) and 77 samples for dickkopf‐1 (catalog no. DKK100B; R&D Systems, Minneapolis, MN, USA). Analysis was stopped when significant associations were revealed. One Ocn and one DKK1 NOAH data point were excluded from the analysis because of being above their respective reference standard range despite several dilutions. Serum from NHP was also collected by centrifugation before CBA, before SIV infection, and before initiation of ART. The same kits were used to measure Ocn and Scl in these serum samples.

### Micro‐computed tomography

At necropsy, tibias were harvested from each animal, placed into 10% aqueous buffered zinc formalin (Z‐fix), and stored at room temperature before analysis. Tibias were imaged using high‐resolution micro‐computed tomography (VivaCT 40, Scanco Medical, Brüttisellen, Switzerland). Briefly, the proximal tibia and tibial midshaft regions were scanned as 14 μm isotropic voxel size using 70 kVp, 114 mA, and 400 ms. Bone volume fraction (BV/TV, %), trabecular thickness (Tb.Th, mm), trabecular separation (Tb.Sp, mm), trabecular number (Tb.N, mm^−1^), connectivity density (ConnD, mm^−3^), and structure model index (SMI) were calculated using previously published methods.^(^ ^39^ ^)^ The cancellous bone region was obtained using a semiautomated contouring program that separated cancellous from cortical bone. At the midshaft of the tibia, total cross‐sectional area (CSA, mm^2^), medullary area (MA, mm^2^), and cortical thickness (Ct.Th, mm) were assessed in a 1‐mm‐long region centered at the midshaft. Bone was segmented from soft tissue using the same threshold for all groups, 245 mg HA/cm^3^ for trabecular and 682 mg HA/cm^3^ for cortical bone. All micro‐CT scanning and analyses were compliant with American Society for Bone and Mineral Research (ASBMR) guidelines.^(^ ^40^ ^)^

### Data analysis

Statistical analysis was performed using GraphPad Prism v.9.3.1 (GraphPad, Inc., San Diego, CA, USA). A reciprocal model provided a superior fit to the curvature of data sets plotting markers of bone formation against measures of alcohol use compared with other standard models; therefore, Ocn and PINP values were converted to their reciprocals to allow for multiple linear regression analysis. Pearson coefficients (*r*) and corresponding *p* values are presented when the relationship between the two variables is reasonably linear; otherwise, Spearman (*r*_s_) correlations are shown. Seven covariates (age, sex, education, race, smoking status, viral load, and cluster of differentiation‐4 [CD4] count) were adjusted for in multivariate linear regression analyses based on known association with alcohol and bone health, as well as preliminary analyses of alcohol consumption in the study population. Fully adjusted models included other measures of alcohol as additional covariates. When evaluating CBA and OVX effects on trabecular and cortical parameters, two‐way ANOVA analyses were performed.

## Results

### Ocn and PINP are inversely associated with measures of alcohol consumption

**Fig. 1:**
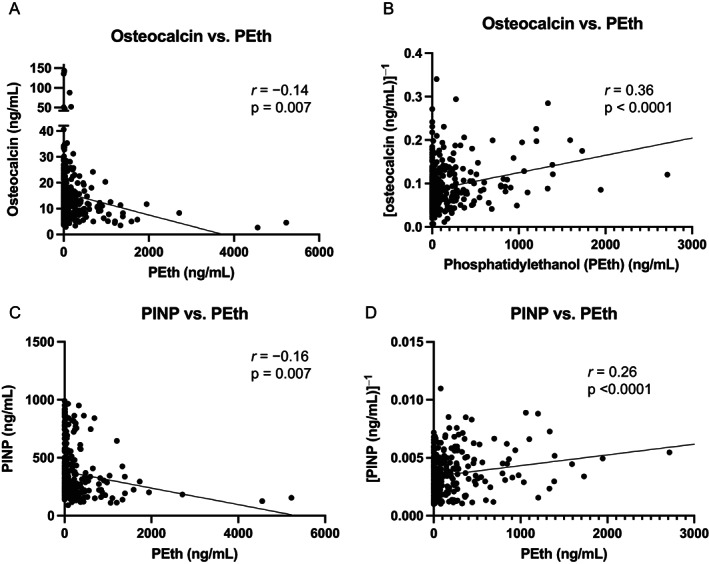
Osteocalcin, pro‐collagen I N‐terminal propeptide (PINP), and their reciprocal plotted against phosphatidylethanol. (*A*) Circulating osteocalcin levels are plotted against phosphatidylethanol (PEth). (*B*) Reciprocal transformation of osteocalcin values was performed for multiple linear regression analysis. Three data points are outside the axis limits because of *y* axis shortening for clarity. Each data point represents 1 participant (*n* = 349). (*C*) Similarly, circulating PINP levels are plotted against PEth (*n* = 356), and (*D*) the reciprocal of PINP is plotted against PEth.

Characteristics of our NOAH cohort sample (*n* = 356), whose patients are predominantly male (69.9%), Black (83.4%), and over 40 years old (77.8%), are reported in Table 1. We found that Ocn concentrations were inversely associated with the various measures of alcohol use (Fig. 1). Ocn was subsequently transformed to its reciprocal to allow for simple linear regression analyses (Table 2) and evaluation of monotonic relationships (Supplemental Table S1). Ocn was best correlated with phosphatidylethanol (PEth, *r* = 0.36, *p* < 0.0001; Fig. 1), followed by TLFB at days 14 and 30 (TLFB14 and TLFB30, *r* = 0.25, *p* = <0.0001), and AUDIT‐C (*r* = 0.1821, *p* = 0.0006) (Supplemental Table S1). Ocn was not correlated with either the total AUDIT score or with lifetime drinking history (LDH) (Supplemental Table S1). Adjusting for the seven biological and socioeconomic factors (age, sex, education, race, smoking status, viral load, and CD4 count) generally increased the coefficients of regression between Ocn and measures of alcohol consumption and decreased associated *p* values (Table 2). When all measures of alcohol use were included in the regression model, AUDIT‐C and LDH provided no statistically significant explanatory power beyond PEth and TLFB14 (Table 2).

**Table 1:** Characteristics of the Studied NOAH Sample (*n* = 356) and the Subsample Studied for Sclerostin (*n* = 154)

	Full study (*n* = 356)	Sclerostin sample (*n* = 154)	*p* value
	% (*n*)		
Sex			0.966
Men	69.9 (249)	70.1 (108)	
Women	30.1 (107)	29.9 (46)	
Education			0.859
<High school	40.2 (143)	40.9 (63)	
High school graduate	31.7 (113)	29.9 (46)	
Some college	22.2 (79)	21.4 (33)	
College graduate	5.9 (21)	7.8 (12)	
Race			0.775
Black	83.4 (297)	81.2 (125)	
White	15.7 (56)	18.2 (28)	
Other	0.8 (3)	0.7 (1)	
Smoking status			0.813
Never smoker	23.0 (82)	20.8 (32)	
Former smoker	16.6 (59)	18.2 (28)	
Current smoker	60.4 (215)	61.0 (94)	
Age (years)			0.897
20–29	5.3 (19)	4.6 (7)	
30–39	16.9 (60)	17.5 (27)	
40–49	23.6 (84)	21.4 (33)	
50–59	41.6 (148)	40.9 (63)	
60+	12.6 (45)	15.6 (24)	
Viral load (copies/mL)			0.516
<20	69.2 (245)	72.1 (111)	
≥20	30.8 (109)	27.9 (43)	
CD4 count (cells/μL)			0.537
<200	13.3 (47)	16.9 (26)	
200–350	13.3 (47)	11.7 (18)	
>350	73.5 (260)	71.4 (110)	
	Median (IQR)		
Alcohol measures			
PEth (ng/mL)	34.0 (179.0)	34.0 (146.0)	1
AUDIT score	6.0 (10.0)	5.0 (8.0)	0.273
Timeline followback (TLFB) total grams	166.6 (753.9)	147.0 (647.4)	0.779
TLFB, 30 days (no. of d/30 days)	3.0 (12.0)	2.5 (11.0)	0.658
TLFB, 14 days (no. of d/14 days)	2.0 (6.0)	2.0 (6.0)	1

*Note*: The sample studied for sclerostin did not significantly differ from the full study cohort in any parameter.

Abbreviations: AUDIT, Alcohol Use Disorder Identification Test; NOAH, New Orleans Alcohol Use in HIV study; PEth, phosphatidylethanol.

**Table 2:** Multiple Linear Regression of Serum Osteocalcin Versus Alcohol Consumption Measures

Mean: 15.29 ng/mL	[Serum osteocalcin (ng/mL)]^−1^					
Median: 12.05 ng/mL	Crude model		Model adjusted for:			
Range: 141.2 ng/mL			Age, sex, education, race, smoking status, viral load, and CD4 count		+ measures of ethanol use (PEth, TLFB14, AUDIT‐C, LDH)	
IQR: 8.90 ng/mL						
*n* = 355						
	β	*p* Value	β	*p* Value	β	*p* Value
PEth (ng/mL)	5.5 × 10^−6^	<0.0001	4.3 × 10^−5^	<0.0001	3.7 × 10^−5^	<0.0001
TLFB (g/14 days)	4.5 × 10^−6^	<0.0001	2.3 × 10^−5^	<0.0001	1.2 × 10^−6^	0.0231
AUDIT‐C	2.9 × 10^−3^	0.0006	3.4 × 10^−3^	<0.0001	2.8 × 10^−4^	0.7941
LDH (kg)	6.7 × 10^−6^	0.2971	1.2 × 10^−5^	0.0821	2.5 × 10^−6^	0.7371

*Note*: Descriptive statistics of serum osteocalcin are listed in the left panel. The first pair of data columns represent the crude (unadjusted) linear regressions between osteocalcin and each of the four listed measures of alcohol use (PEth, TLFB14, AUDIT‐C, LDH). These measures were chosen as they assess alcohol use over different timespans (PEth: 2–3 weeks; TLFB14: 14 days; AUDIT‐C: 1 year; LDH: lifetime). The second pair of columns adjust the regression for the seven listed covariates. Sex and race significantly emerged as significant covariates (not shown in table) and viral load was a significant contributor in the regression between osteocalcin and PEth (not shown in table). The third set of columns further adjusts the model for the other three measures of alcohol (eg, the PEth model is further adjusted for TLFB14, AUDIT‐C, and LDH).

Abbreviations: AUDIT‐C, Alcohol Use Disorder Identification Test (first three items); LDH, lifetime drinking history; PEth, phosphatidylethanol; TLFB14, timeline followback day 14.

Serum PINP, the designated reference marker of bone formation in osteoporosis, was measured to examine how it related to the commonly used Ocn and whether it correlated better with measures of alcohol consumption. Ocn and PINP were highly correlated (*r*_s_ = 0.64, *p* < 0.0001) though not linearly related (Supplemental Fig. S2). Like Ocn, PINP was inversely related to markers of alcohol consumption, with a weaker correlation with PEth (*r* = 0.26 versus *r* = 0.3647) but slightly enhanced coefficients with all self‐reported measures (Supplemental Table S2).

### Biological variables affect the relationship between alcohol intake and Ocn

**Fig. 2:**
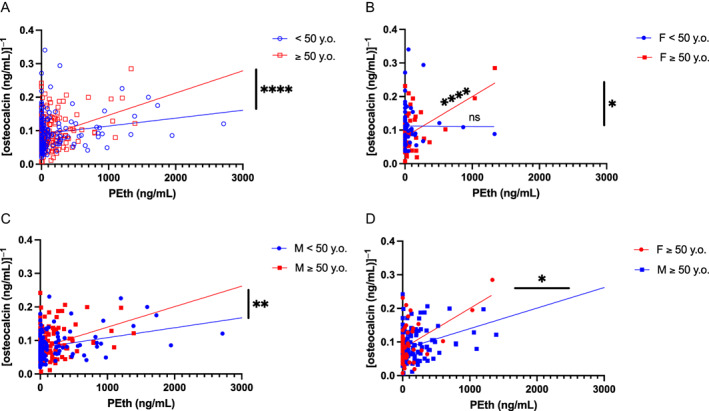
Osteocalcin (Ocn) versus phosphatidylethanol (PEth) by age and sex. The ß coefficients and intersections of the regression between Ocn and PEth were compared by biological variables. (*A*) The ß coefficient is significantly higher in New Orleans Alcohol Use in HIV (NOAH) patients ≥50 years old. (*B*) There is no relationship between PEth and Ocn in females <50 years but a significant one in females ≥50 years. (*C*) Males ≥50 years have a steeper slope of Ocn decline with PEth levels, and (*D*) females ≥50 years have significantly lower levels of Ocn with increasing PEth levels than their male counterparts of the same age. Two data points are excluded from each graph because of axis shortening for clarity. *n =* 349. *p ≤ 0.05; **p ≤ 0.01; **** *p* ≤ 0.0001.

Regression modeling demonstrated that patients over the age of 50 years had steeper decreases in Ocn levels with PEth increases compared with younger patients (*p* < 0.05; Fig. 2*A*).Females over 50 years demonstrated the steepest declines in Ocn with rising levels of PEth of all groups, whereas in younger females, there was no relationship between these two variables (F <50 years, ß = −1.324 × 10^−6^, *p* = 0.9734; F ≥50 years, ß = 1.195 × 10^−4^, *p* = 0.0001) (Fig. 2*B*). Of note, because the slopes significantly differed, it is not possible to compare the intercepts, though F ≥50 years had a smaller intercept. Compared with men in the same age category, females over 50 years had significantly more precipitous reductions in osteocalcin in function of PEth (Fig. 2*D*). Men ≥50 years also had sharper declines in Ocn with PEth than their younger counterpart (*p* = 0.0021; Fig. 2*C*).

Neither sex or race significantly altered regression coefficients, though female and Black patients overall have significantly lower levels of Ocn than their counterparts at any PEth level (different intercepts *p* < 0.05; not shown). Viral load was also linked to less Ocn when adjusting for PEth but not when adjusting for other EtOH measures (not shown). Smoking status, age, CD4 count, and education were not found to influence circulating levels of Ocn.

### Scl, but not DKK1, is positively associated with measures of alcohol use and negatively with Ocn

**Fig. 3:**
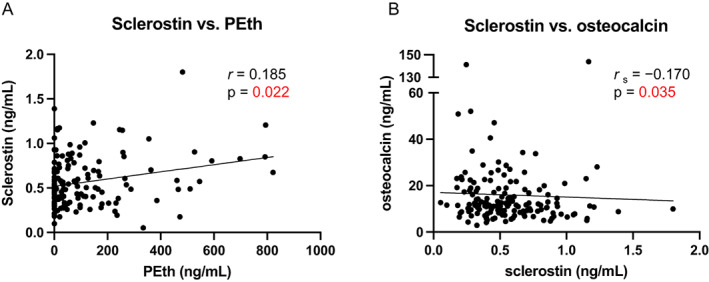
Sclerostin (Scl) is positively correlated to phosphatidylethanol (PEth) and negatively with osteocalcin (Ocn). Circulating Scl is plotted against (*A*) PEth (*n =* 153) and (*B*) osteocalcin (*n* = 153). Solid line = linear trend; *r* = Pearson correlation coefficient; *r*_s_ = Spearman correlation coefficient; p = associated *p* value.

To obtain mechanistic insights, we measured serum sclerostin (Scl) and dickkopf‐1 (DKK1), both osteocyte‐derived negative regulators of bone formation. Analyzing 42% of the cohort (*n* = 154) was sufficient to reveal a positive correlation between serum Scl and both PEth (*r*_s_ = 0.185, *p* = 0.022; Fig. 3) and TLFB30 (*r*_s_ = 0.165, *p* = 0.041; not shown). The distributions of the variables in the subsample studied for sclerostin reflected the distributions of each variable in the full study population (Table 1). The correlation coefficient was similar with TLFB14 but did not reach significance (*r*_s_ = 0.155, *p* = 0.055) and was much weaker with AUDIT, AUDIT‐C, and LDH (*r*_s_ = [0.115, 0.132], n.s. = not shown). To uncover whether Scl was linked to inhibition of bone formation, we examined the relationship between Scl and both Ocn and PINP. Circulating levels of Ocn and Scl negatively correlated (*r*_s_ = −0.170, *p* = 0.035) as opposed to those of Scl and PINP, which did not. Adjusting for covariates (sex, race, age, viral load, CD4 count, education, and smoking status) generally attenuated the coefficients and increased the *p* values of the regressions between Scl and the various measures of alcohol consumption (Supplemental Table S3), leaving only the association between Scl and PEth significant (*p* = 0.0043). Age was associated with increased Scl (not shown). Further, adjusting for Scl eliminated the significance of the association between PEth and Ocn (Table 3). DKK1 did not display significant associations to PEth (Supplemental Fig. S2 *A*, *B*).

**Table 3:** Adjusting for Sclerostin Annuls the Regression Between Ocn and PEth

Mean: 15.29 ng/mL	[Serum osteocalcin (ng/mL)]^−1^			
Median: 12.05 ng/mL	Crude model		Model adjusted for:	
Range: 141.2 ng/mL			+ sclerostin	
IQR: 8.90 ng/mL				
*n* = 355				
	β	*p* value	β	*p* value
PEth (ng/mL)	5.5 × 10^−6^	<0.0001	4.0 × 10^−5^	0.1856
TLFB (g/14 days)	4.5 × 10^−6^	<0.0001	−2.8 × 10^−6^	0.842
AUDIT‐C	2.9 × 10^−3^	0.0006	4.2 × 10^−4^	0.83
LDH (kg)	6.7 × 10^−6^	0.2971	−6.4 × 10^−6^	0.6361

*Note*: The first set of columns show the ß coefficients and associated *p* values of simple linear regression between Ocn and listed measures of alcohol. The second pair of columns show the model adjusted for seven previously listed covariates (age, sex, education, race, smoking status, viral load, and cluster of differentiation‐4 [CD4] count), measures of alcohol (PEth, TLFB14, AUDIT‐C, and LDH) as well as sclerostin.

Abbreviations: AUDIT‐C, Alcohol Use Disorder Identification Test (first three items); LDH, lifetime drinking history; Ocn, osteocalcin; PEth, phosphatidylethanol; TLFB, timeline followback.

### OVX and CBA both affect tibial trabecular parameters, but only OVX affects cortical bone in female non‐human primates (NHP)

**Fig. 4:**
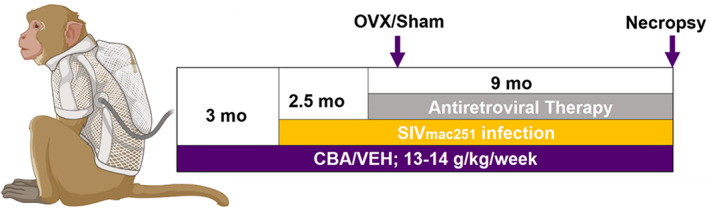
Visual representation of non‐human primate study design.

**Fig. 5:**
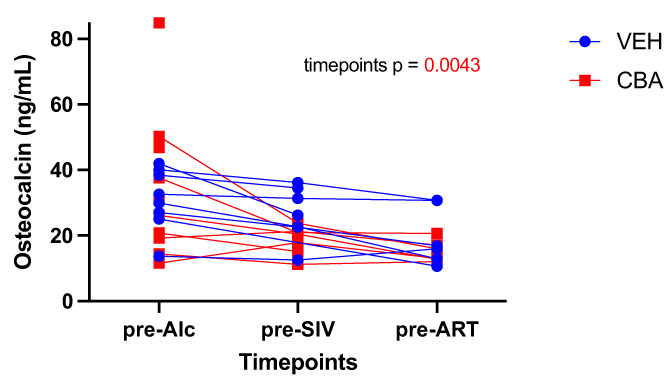
Serum osteocalcin in related simian immunodeficiency virus (SIV)‐infected non‐human primate (NHP) cohort. NHP serum was collected before inception of chronic binge alcohol (CBA)/vehicle (VEH) treatment (pre‐Alc), 2.5 months later before SIV inoculation (pre‐SIV) and 3 months later before antiretroviral therapy (ART) (pre‐ART). CBA and SIV did not significantly change Ocn levels per repeated measures ANOVA, although time points emerged significant. The pre‐Alc 84.91 ng/mL data point in the CBA group represents an outlier in this pre‐Alc data set, the pre‐Alc *n* = 8 (VEH), 10 (CBA); pre‐SIV *n =* 7 (both groups); pre‐ART *n =* 6 (both groups).

We next measured serum Ocn in the NHP model (Fig. 4) to confirm the expected CBA‐dependent decrease in Ocn and to dissociate the effects of HIV and ART from those of alcohol. Results showed alcohol‐ and time point–dependent decreases in Ocn, with only time point being significant per mixed‐effect analysis (*p* = 0.0047; Fig. 5).

**Fig. 6:**
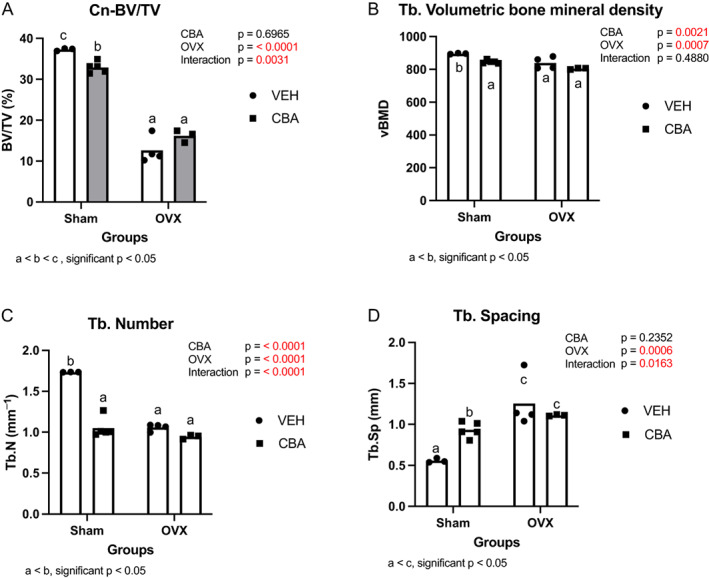
Tibial trabecular (Tb) parameters are affected by both chronic binge alcohol (CBA) and ovariectomy (OVX). (*A*) OVX approximately halves bone volume fraction (BV/TV), whereas CBA has no effect on this parameter. (*B*) Vehicle (VEH) and CBA both decrease volumetric bone mineral density (vBMD). (*C*) OVX and CBA both similarly reduce trabecular number by approximately 50%, with no additive effects of the treatments. (*D*) OVX significantly increases trabecular spacing. Turkey post hoc comparisons are shown. Data are mean with individual data points. *n =* 3 for VEH/Sham group; *n =* 4 for VEH/OVX group; *n =* 5 for CBA/Sham group; *n =* 3 for CBA/OVX group.

**Fig. 7:**
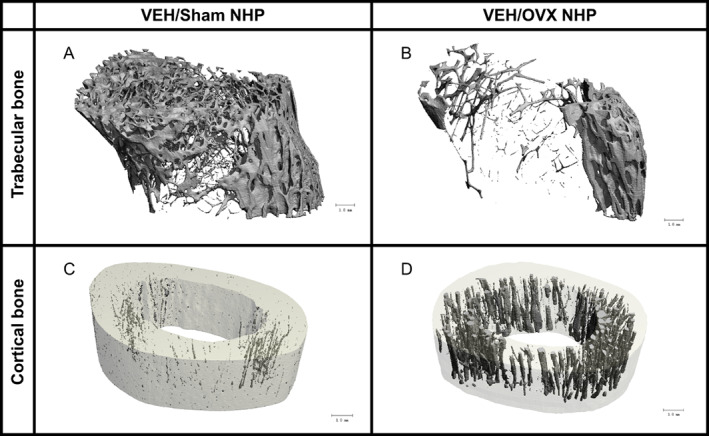
Three‐dimensional reconstruction of trabecular (*A*, *B*) and cortical (*C*, *D*) tibial bone from one vehicle (VEH)/Sham (*A–C*) and one VEH/ovariectomy (OVX) (*B–D*) group‐representative non‐human primate (NHP). OVX has substantial effects on both trabecular and cortical bone, whereas CBA mostly impacts trabecular bone (not shown).

Micro‐CT results from the NHP bones at the end of combined SIV, ART, and CBA exposure in intact and OVX monkeys are presented in Fig. 6 (trabecular parameters), Table 4 (cortical parameters), and Fig. 7 (3D reconstructions of representative samples from VEH/Sham and VEH/OVX groups). OVX decreased BV/TV, volumetric bone mineral density (vBMD), and Tb.N and increased Tb.Sp (*p* < 0.05) (Fig. 6), with no impact on Tb.Th (not shown). CBA similarly impacted only two of these parameters, decreasing vBMD (*p* = 0.0021) and Tb.N (*p* < 0.0001) but having no effect on BV/TV. Two‐way ANOVA analysis revealed CBA significantly interacted with OVX to alter BV/TV, Tb.N, and Tb.Sp, with CBA dampening additional OVX effects on trabecular number and spacing. OVX impacted cortical bone, diminishing cortical thickness and increasing cortical porosity (*p* < 0.0001). CBA alone significantly decreased marrow area, especially in the OVX group (*p* < 0.0001).

**Table 4:** Tibial Cortical (Ct) Parameters Are Mainly Affected by OVX: OVX Decreases Cortical Thickness and Area and Increases Porosity

	Cortical thickness	Cortical porosity	Cortical area	Periosteal perimeter	Endosteal perimeter	Marrow area
CBA	ns	ns	ns	ns	ns	↓ 1.5%***
OVX	↓ 21.8%***	↑ 421.2%***	↓ 1.8%**	ns	ns	↑ 7.3%*
Interaction	ns	ns	ns	ns	ns	***

*Note*: CBA has no effect on these parameters. Marrow area is decreased by CBA and increased by OVX, with significant CBA/OVX interaction on this parameter. *n* = 4 for VEH/Sham group; *n* = 4 for VEH/OVX group; *n* = 5 for CBA/Sham group; *n* = 3 for CBA/OVX group.

Abbreviations: CBA, chronic binge alcohol; OVX, ovariectomy.

* *p* ≤ 0.05.

** *p* ≤ 0.001.

*** *p* ≤ 0.0001.

## Discussion

There is a widespread notion that light alcohol consumption carries a variety of health benefits, from it being tied to lower blood pressures and risk of cardiovascular events and decreased risk of infections, peptic disease, and even gallstone formation.^(^ ^41^ ^)^ Whether this J‐shaped curve of reduced relative risk for disease with light consumption applies to bone, particularly in PLWH, remains unclear. In this study, we analyzed serum samples of 356 PLWH enrolled in the NOAH study and found that Ocn and PINP are both inversely associated with multiple assessments of both self‐report and biochemically determined alcohol consumption. These findings strongly suggest that any level of alcohol consumption is linked to lessened osteoblast function. Most epidemiological data suggesting that low alcohol consumption is bone protective is obtained from postmenopausal women.^(^ ^42^, ^43^ ^)^ Menopause is associated with an increased rate and frequency of bone turnover, and therefore it is possible that alcohol dampens that acceleration, resulting in heightened bone mass in light drinkers. Alternatively, the clinical data could suggest a greater sensitivity to deleterious effects of alcohol in PLWH.

The strongest correlation existed between Ocn and PEth, presumably related to the obvious limitations in self‐report measures. Ocn did not correlate with total AUDIT score and LDH, suggesting that circulating Ocn fluctuates in function of recent consumption and suppression of bone formation is at least partly reversible upon termination of drinking.^(^ ^44^, ^45^ ^)^ Limitations of PEth as a marker is that it is simultaneously not reflective of drinking pattern and carries a long half‐life (4 days), and there is potential overlap in concentrations between moderate and heavy drinkers (it is not validated as a continuous variable).^(^ ^46^, ^47^, ^48^ ^)^ Nevertheless, it seems unlikely that any one marker of alcohol use would strongly correlate with a single bone‐derived factor as it is widely accepted that alcohol's skeletal toxicity is multifaceted.^(^ ^49^, ^50^ ^)^

Ocn is a small (49‐amino‐acid) noncollagenous protein hormone that serves as an important bone‐specific factor, particularly in its uncarboxylated form. Its endocrine roles in energy metabolism, neural development, muscle growth, and male fertility are believed to be greater than those in bone mineralization and bone density.^(^ ^51^ ^)^ In contrast, PINP is a peptide derived from posttranslational cleavage of type I procollagen molecules and is the most specific and sensitive marker of bone formation, especially helpful in monitoring bone health.^(^ ^52^ ^)^ Therefore, circulating concentrations of PINP directly reflect the rate of type I collagen synthesis, which constitutes around 90% of the organic bone matrix.^(^ ^53^ ^)^ Given the stronger regression coefficients of PEth with Ocn compared with PINP, we propose that Ocn may act as a more reliable indicator of alcohol's impact on bone function globally.

Not surprisingly, age emerged as a strong predisposing factor, with patients aged ≥50 years experiencing sharper declines in Ocn with drinking. This trend applied to both men and women, though interestingly, females over 50 years experienced more precipitous declines in Ocn with increased PEth levels than their male counterparts, and there was no apparent relationship between Ocn and PEth in women younger than 50 years. Though this could be attributable to small sample size in the young females, unchanged osteoblast function with alcohol exposure in a premenopausal setting supports a protective role of estradiol against the bone‐detrimental effects of alcohol exposure, perhaps even in men.^(^ ^54^, ^55^, ^56^ ^)^ Further, our data suggest that gonadal insufficiency is an additional contributing factor to susceptibility beyond aging. The average age of menopause onset in the United States is 51 years and the global range is 48 to 51 years.^(^ ^25^ ^)^ With menopause, the rate of bone loss increases from 3% to 7% per year due to heightened rate of remodeling with a net increase in bone resorption.^(^ ^22^, ^23^, ^24^ ^)^ Regardless of how alcohol impacts remodeling rate in postmenopausal women, there seems to be a clear inhibition of bone formation tied to any amount of alcohol consumption in female PLWH. Women and Black patients had significantly lower Ocn levels at any given PEth level, concordant with a previous study characterizing patterns of Ocn expression across age, sex, and ethnic groups.^(^ ^57^ ^)^ Black individuals have a higher bone density than non‐Hispanic Whites, an explanation for which might be that turnover is overall lower but the formation‐to‐resorption ratio still greater in Blacks.^(^ ^58^, ^59^ ^)^

Sclerostin (Scl), the product of the *Sost* gene, is a potent antagonist of the bone anabolic Wnt/ß‐catenin signaling pathway.^(^ ^29^ ^)^ Chronic alcohol feeding represses mediators of this pathway in bone,^(^ ^50^ ^)^ and HIV infection has been linked to Wnt/ß‐catenin signaling inhibition in primary human osteoblasts other tissues.^(^ ^60^, ^61^ ^)^ We hypothesized that Scl would positively correlate with alcohol use and negatively with biomarkers of bone formation. We report that serum Scl levels positively correlate with PEth in a fully adjusted model including age as a covariate, which is related to increased Scl levels.^(^ ^62^ ^)^ Scl has previously been reported to be negatively associated with Ocn but not with nutritional status or alcohol intake.^(^ ^63^ ^)^ Adjusting for Scl rendered the interaction between Ocn and PEth nonsignificant, supporting a link between circulating Scl and the pathogenesis of alcohol‐induced osteopenia. The lack of association between Scl and PINP, the current preferred clinical marker of bone formation in testing for osteoporosis, is consistent with a previous report,^(^ ^64^ ^)^ as well as a more global impact of sclerostin on osteoblasts. Interestingly, Scl correlates positively with BMD in many disease states, including in PLWH although independently of HIV‐serostatus.^(^ ^64^, ^65^, ^66^ ^)^ A new study suggests that raised Scl levels are also related to fat deposition and increased body mass index (BMI) regardless of liver function.^(^ ^67^ ^)^ Alcohol promotes differentiation of bone marrow mesenchymal stem cells toward the adipogenic over osteoblastic lineage.^(^ ^68^ ^)^ Mechanistic studies are required to assert a causative action of this Wnt antagonist. Importantly, circulating Scl levels might not accurately reflect changes in local production of sclerostin in the bones in response to alcohol.^(^ ^69^ ^)^

Dickkopf‐1 (DKK1) is another Wnt antagonist secreted by osteocytes, although it is not specific to these cells.^(^ ^70^ ^)^ DKK1 has been involved in the pathogenesis of multiple myeloma, femoral head necrosis, and obesity.^(^ ^66^, ^71^, ^72^, ^73^ ^)^ Unlike Scl, it tends to be negatively associated with BMD.^(^ ^66^ ^)^ Because alcohol use is associated with both inhibition of Wnt/ß‐catenin signaling^(^ ^50^ ^)^ and osteocyte apoptosis,^(^ ^74^, ^75^ ^)^ we measured their circulating levels to assess potential alcohol‐dependent fluctuations. No significant associations were observed between DKK1 and PEth; however, it is possible that the sample size was insufficient to detect a significant association.

The female NHP model provided a bidirectional translational approach to validate our clinical cohort findings by extending them to a controlled animal model. In this model, we examined alcohol‐exposed bone and whether gonadal loss exacerbates CBA's effects on the skeleton. We measured serum Ocn levels before OVX and found time‐ and alcohol‐dependent decreases in this marker. No discernible trend was visible with Scl (not shown). Because most the available samples were obtained before initiation of CBA/VEH treatment (baseline) and a limited number of samples were available from later time points, we attribute the nonsignificance of our findings to a low sample number and halted any continuing serum analysis for this reason. It is likely that more samples would establish a clear relationship between Ocn and CBA based on the literature and our own clinical cohort findings. The effect of SIV on circulating Ocn remains uncertain; of note, HIV is established as resorption promoting rather than formation inhibiting.^(^ ^13^ ^)^

Tibial histomorphometric analysis revealed striking independent results for both CBA and OVX, which both decreased trabecular number rather than thickness to increase spacing. OVX, in contrast to CBA, also significantly decreased trabecular bone fraction. The magnitude of OVX effects on trabecular number and spacing was damped by previous and/or concurrent CBA treatment. These findings along with the clinical data suggest that in premenopausal women, no net change in total osteoblast function (bone volume fraction) could still be accompanied by significant bone microstructural changes (trabeculae) and decreases in mineralization, and therefore, decreased bone strength. Likewise, it is plausible that despite apparent plateaued effects of CBA on certain trabecular parameters, CBA/OVX bone might be more fragile than CBA/VEH bone. Only OVX seems to have profound effects on intracortical remodeling, at least in female rhesus macaques. Interestingly, Gaddini and colleagues performed a 12‐month study of voluntary heavy alcohol consumption in male *Macaca mulatta* and also found that cortical thickness was not impacted by CBA. However, they reported CBA‐dependent decreased cortical porosity in the tibial midshaft as well as negatively impacted osteon density.^(^ ^76^ ^)^ Although CBA did not emerge as a significant parameter in our analysis, cortical porosity was decreased 7% in the CBA/OVX group compared with Sham/OVX, with a trending interaction (*p* = 0.0666) between CBA and OVX. A few possible explanations could reconcile these divergent findings, including the different patterns of drinking of the studies and a sex difference attributable to an estradiol‐protective effect on alcohol interference with cortical remodeling. A CBA‐dependent diminished labeled osteon density in male NHP suggests alcohol interference with perilacunar‐canalicular remodeling (PLR) in males, a process that would be worth assessing in females given that PLR‐mediated bone quality influences fracture risk.^(^ ^77^, ^78^ ^)^

This study reports several notable findings. Raised alcohol levels as per several measures of alcohol use are tied to precipitous declines in both Ocn and PINP levels, indicating an inhibition of osteoblast function at any level of alcohol use in PLWH. Age and female gonadal loss each appear to increase sensitivity of osteoblasts to alcohol effects, consistent with a heightened susceptibility to osteoporosis in postmenopausal women. Circulating Scl appears related in some way to the pathogenesis of alcohol‐induced osteopenia; whether as a reliable marker or mediator of disease remains to be determined. NHP data in view of clinical findings support that CBA may significantly alter bone morphology without a net apparent decline in osteoblast function. Limitations include lack of BMD measurements or fracture risk assessment (FRAX tool). This investigation warrants continuation with inclusion of sophisticated bone health assessments and comparison with HIV‐seronegative and HIV‐positive, ART naïve cohorts. Future studies in NHPs should continue to characterize alcohol‐dependent changes in bone microarchitecture and investigate sexual dimorphism in local bone responses to alcohol, including alterations in expression of *Sost*/sclerostin and other osteocyte‐derived factors, and relate them to serum findings. A deeper understanding of the interplay between alcohol, HIV, and ART and biological variables on bone disease will inform therapeutic strategies and interventions for PLWH.

## Author Contributions

**Alexandra Denys:** Conceptualization; data curation; formal analysis; funding acquisition; investigation; writing – original draft. **Allison Norman:** Data curation; writing – review and editing. **Daniel S Perrien:** Investigation; methodology; writing – review and editing. **Larry J Suva:** Data curation; investigation; writing – review and editing. **Liz Simon:** Funding acquisition; investigation; project administration; writing – review and editing. **Lee S McDaniel:** Data curation; validation; writing – review and editing. **Tekeda Ferguson:** Data curation; funding acquisition; project administration; validation; writing – review and editing. **Kim Pedersen:** Conceptualization; formal analysis; writing – review and editing. **David Welsh:** Funding acquisition; investigation; project administration; writing – review and editing. **Patricia E Molina:** Funding acquisition; project administration; writing – review and editing. **Martin JJ Ronis:** Conceptualization; funding acquisition; investigation; project administration; supervision; writing – review and editing.

## Conflict of interest

The authors have no conflicts of interest.

### Peer Review

The peer review history for this article is available at https://publons.com/publon/10.1002/jbm4.10703.

## Supporting information


**Supplemental Fig. S1.** Temporally related measures of alcohol use are highly correlated. The inferior left portion of the heat map displays Spearman correlation coefficients between measures of alcohol use, while the superior right shows the Pearson correlation coefficients.

**Supplemental Fig. S2.** Serum osteocalcin and pro‐collagen I N‐terminal propeptide (PINP) are highly correlated. Loss of linearity between osteocalcin and PINP is observed above approximately 25 ng/mL of osteocalcin. *n =* 355.

**Supplemental Fig. S3.** Dickoppf‐1 versus phosphatidylethanol. PEth plotted against dickkopf‐1 (*n =* 74).

**Supplemental Table S1.** Correlation between serrum Ocn and related alcohol use measures.

**Supplemental Table S2.** Correlation between serum PINP and related alcohol use measures.

**Supplemental Table S3.** Correlation between serum sclerostin and related alcohol use measures.
