# Supplementary figures and images for: Impact of Alcohol on Bone Health in People Living With HIV: Integrating Clinical Data From Serum Bone Markers With Morphometric Analysis in a Non‐Human Primate Model
Source: JBMR Plus. 2022 Nov 28;7(1):e10703. doi: 10.1002/jbm4.10703 (PMC9850440; doi:10.1002/jbm4.10703)

## Slide 1
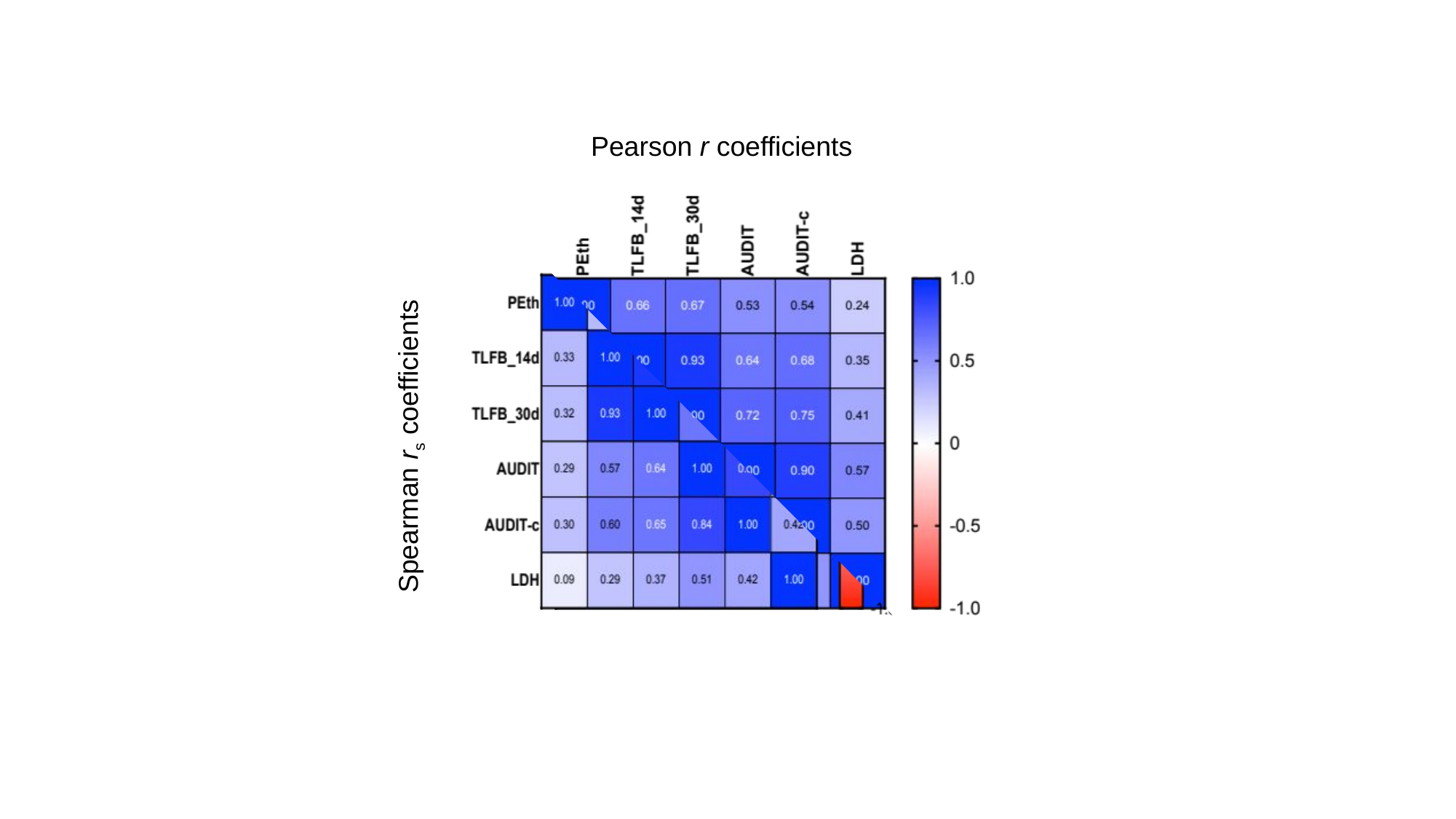

Pearson r coefficients
Spearman rs coefficients

Supplement: Supplementary file 1 — Supplemental Fig. S1. Temporally related measures of alcohol use are highly correlated. The inferior left portion of the heat map displays Spearman correlation coefficients between measures of alcohol use, while the superior right shows the Pearson correlation coefficients. [file JBM4-7-e10703-s001.pptx]

## Slide 1
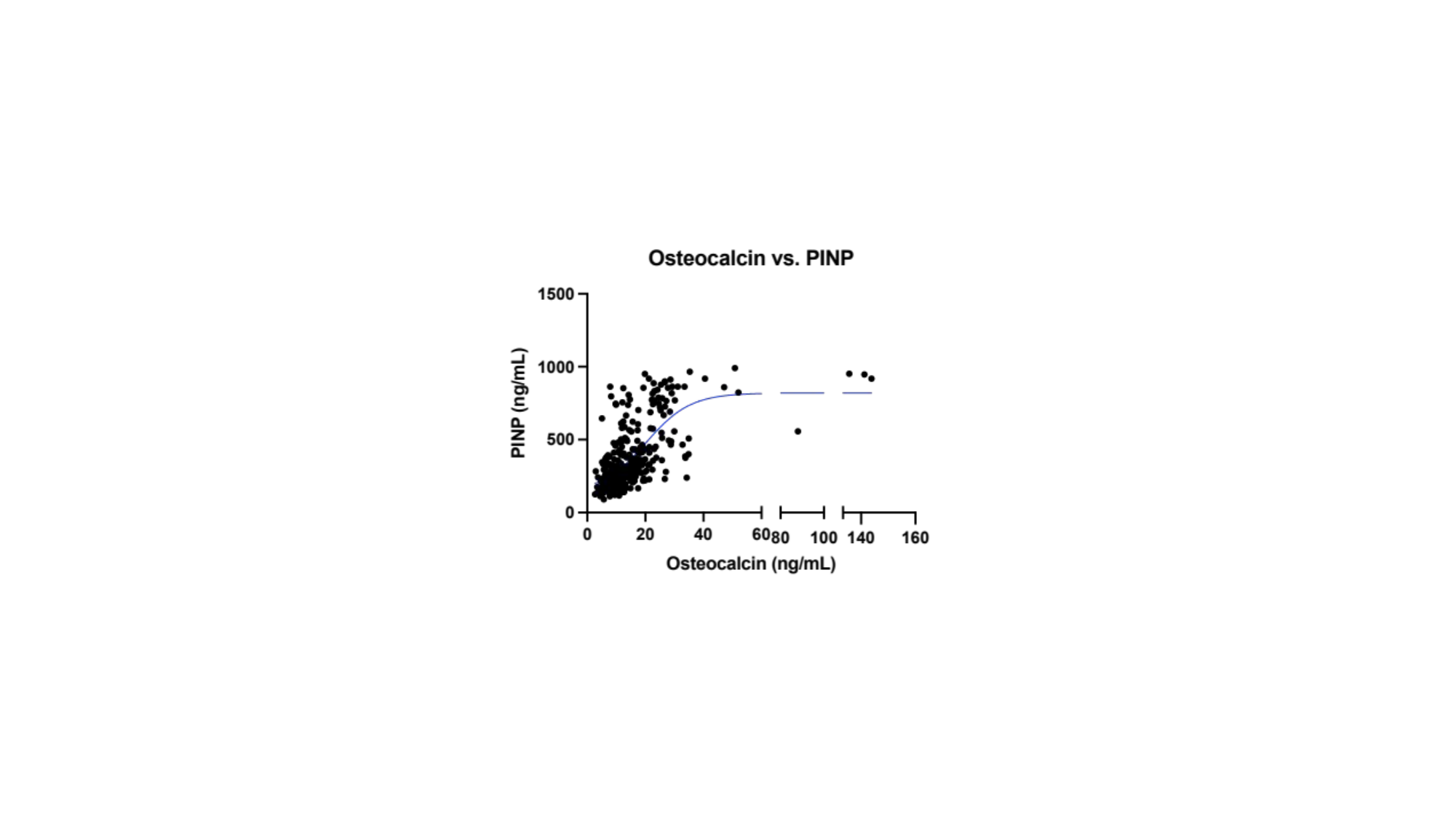

Supplement: Supplementary file 2 — Supplemental Fig. S2. Serum osteocalcin and pro‐collagen I N‐terminal propeptide (PINP) are highly correlated. Loss of linearity between osteocalcin and PINP is observed above approximately 25 ng/mL of osteocalcin. n = 355. [file JBM4-7-e10703-s002.pptx]

## Slide 1
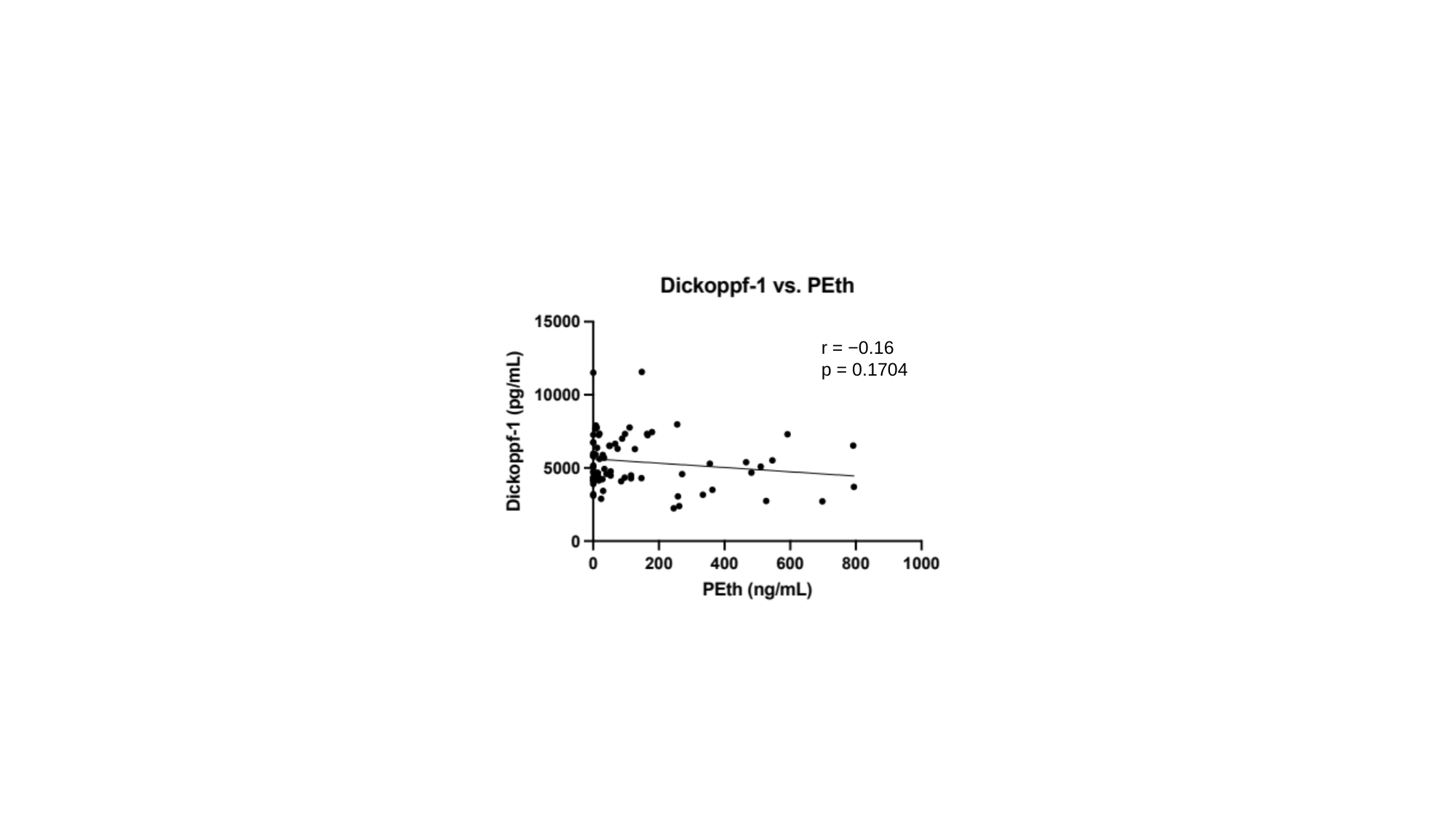

r = −0.16
p = 0.1704

Supplement: Supplementary file 3 — Supplemental Fig. S3. Dickoppf‐1 versus phosphatidylethanol. PEth plotted against dickkopf‐1 (n = 74). [file JBM4-7-e10703-s006.pptx]
