# Supplementary material for: Impact of Alcohol on Bone Health in People Living With HIV: Integrating Clinical Data From Serum Bone Markers With Morphometric Analysis in a Non‐Human Primate Model
Source: JBMR Plus. 2022 Nov 28;7(1):e10703. doi: 10.1002/jbm4.10703 (PMC9850440; doi:10.1002/jbm4.10703)
Supplement: Supplementary file 4 — Supplemental Table S1. Correlation between serrum Ocn and related alcohol use measures. [file JBM4-7-e10703-s004.docx]

|  |  | PEth | TLFB_14d | TLFB_30d | AUDIT-C | AUDIT | LDH |
| --- | --- | --- | --- | --- | --- | --- | --- |
| Ocn | Pearson r | -0.14 | -0.14 | -0.14 | -0.12 | -0.081 | -0.043 |
|  | p value | 0.0072 | 0.0098 | 0.0083 | 0.0198 | 0.129 | 0.4146 |
|  | Spearman r_s_ | -0.21 | -0.22 | -0.22 | -0.2 | -0.16 | -0.044 |
|  | p value | <0.0001 | <0.0001 | <0.0001 | 0.0001 | 0.0029 | 0.4041 |
| Ocn^−1^ | Pearson r | 0.36 | 0.25 | 0.25 | 0.18 | 0.099 | 0.055 |
|  | p value | <0.0001 | <0.0001 | <0.0001 | 0.0006 | 0.0634 | 0.2971 |
|  | n | 349 | 355 | 355 | 355 | 355 | 355 |

**Table S1**
