# Supplementary material for: Impact of Alcohol on Bone Health in People Living With HIV: Integrating Clinical Data From Serum Bone Markers With Morphometric Analysis in a Non‐Human Primate Model
Source: JBMR Plus. 2022 Nov 28;7(1):e10703. doi: 10.1002/jbm4.10703 (PMC9850440; doi:10.1002/jbm4.10703)
Supplement: Supplementary file 5 — Supplemental Table S2. Correlation between serum PINP and related alcohol use measures. [file JBM4-7-e10703-s005.docx]

|  |  | PEth | TLFB_14d | TLFB_30d | AUDIT-C | AUDIT | LDH |
| --- | --- | --- | --- | --- | --- | --- | --- |
| PINP | Pearson r | -0.16 | -0.19 | -0.17 | -0.098 | -0.14 | -0.012 |
|  | p value | 0.0026 | 0.0004 | 0.0014 | 0.0661 | 0.0089 | 0.8245 |
|  | Spearman r_s_ | -0.19 | -0.23 | -0.22 | -0.19 | -0.18 | -0.055 |
|  | p value | 0.0003 | <0.0001 | <0.0001 | 0.0003 | 0.0006 | 0.2996 |
| PINP^−1^ | Pearson r | 0.26 | 0.28 | 0.26 | 0.14 | 0.19 | 0.083 |
|  | p value | <0.0001 | <0.0001 | <0.0001 | 0.0088 | 0.0003 | 0.1197 |
|  | n | 350 | 356 | 356 | 356 | 356 | 356 |

**Table S2**
