# Supplementary material for: Impact of Alcohol on Bone Health in People Living With HIV: Integrating Clinical Data From Serum Bone Markers With Morphometric Analysis in a Non‐Human Primate Model
Source: JBMR Plus. 2022 Nov 28;7(1):e10703. doi: 10.1002/jbm4.10703 (PMC9850440; doi:10.1002/jbm4.10703)
Supplement: Supplementary file 6 — Supplemental Table S3. Correlation between serum sclerostin and related alcohol use measures. [file JBM4-7-e10703-s003.docx]

| ﻿Mean: 0.5611 ng/mL | | Serum sclerostin (ng/mL) | | | |
| --- | --- | --- | --- | --- | --- |
| Median: 0.5059 ng/mL | | Crude model | | Model adjusted for: | |
| Range: 1.748 ng/mL | |  |  | age, gender, education, race, smoking status, viral load, and CD4 count | |
| IQR: 0.374 ng/mL | |  |  |  |  |
| *n* = 154 |  |  |  |  |  |
|  |  | β | p-value | β | p-value |
| ﻿PEth (ng/ml) | | 1.3 × 10^−4^ | 0.0023 | 3.6 × 10^−4^ | 0.0043 |
| TLFB (g/14 d) | | 7.7 × 10^−5^ | 0.1793 | 6.5 × 10^−5^ | 0.2174 |
| TLFB (g/30 d) | | 2.3 × 10^−5^ | 0.0399 | 4.0 × 10^−5^ | 0.0600 |
| AUDIT (total) |  | 4.1 × 10^−3^ | 0.2064 | 4.5 × 10^−3^ | 0.1232 |
| AUDIT-C |  | 0.015 | 0.0478 | 0.013 | 0.0502 |
| LDH (kg) |  | 6.2 × 10^−5^ | 0.0317 | 2.9 × 10^−5^ | 0.6456 |

**Table S3**
